# DEC2 expression is positively correlated with HIF-1 activation and the invasiveness of human osteosarcomas

**DOI:** 10.1186/s13046-015-0135-8

**Published:** 2015-02-28

**Authors:** Tu Hu, Nengbin He, Yunsong Yang, Chengqian Yin, Nianli Sang, Qingcheng Yang

**Affiliations:** Department of Orthopedics, Shanghai Jiao Tong University Affiliated Sixth People’s Hospital, No.600, Yishan Road, Shanghai, 200233 China; Huazhong University of Science and Technology, Wuhan, China; Departments of Biology, Pathology & Laboratory Medicine, Drexel University CoAS, 3245 Chestnut St, PISB 417, Philadelphia, PA 19104 USA; Kimmel Cancer Center, Thomas Jefferson University, Philadelphia, PA USA

**Keywords:** Osteosarcoma, DEC2, SHARP1, HIF-1α, Metastasis, Prognosis

## Abstract

**Background:**

Osteosarcoma is the most common malignancy of bone. HIF-1 (hypoxia-inducible factor 1) activation is critical for the metabolic reprogramming and progression of solid tumors, and DEC2 (differentiated embryonic chondrocyte gene 2) has been recently reported to suppress HIF-1 in human breast and endometrial cancers. However, the roles of HIF-1 and DEC2 in human osteosarcomas remain unclear.

**Methods:**

We evaluated the correlation of DEC2 and HIF-1 expression to the prognosis, and studied the roles of DEC2 and HIF-1 activation in the invasiveness of osteosarcoma. Multiple approaches including immunohistochemical staining of clinical osteosarcoma tissues, siRNA-based knockdown and other molecular biology techniques were used. Particularly, by using a repetitive trans-well culture-based *in vitro* evolution system, we selected a more invasive subpopulation (U2OS-M) of osteosarcoma cells from U2OS and used it as a model to study the roles of DEC2 and HIF-1 in the invasiveness of osteosarcoma.

**Results:**

We found that the expression of DEC2 was positively correlated with HIF-1α levels, and HIF-1α expression positively correlated with poor prognosis in osteosarcomas. DEC2 knockdown in osteosarcoma cell lines (U2OS, MNNG and 143B) attenuated HIF-1α accumulation and impaired the up-regulation of HIF-1 target genes in response to hypoxia. Compared with the low invasive parental U2OS, U2OS-M showed higher levels of DEC2 expression which were confirmed at both mRNA and protein levels. Importantly, we found that the increased DEC2 expression resulted in a more rapid accumulation of HIF-1α in U2OS-M cells in response to hypoxia. Finally, we found that HIF-1 activation is sufficient to upregulate DEC2 expression in osteosarcoma cells.

**Conclusion:**

Taken together, whereas DEC2 was found to promote HIF-1α degradation in other types of tumors, our data indicate that DEC2 facilitates HIF-1α stabilization and promotes HIF-1 activation in osteosarcoma. This implies that DEC2 may contribute to the progression and metastasis of human osteosarcoma by sensitizing tumor cells to hypoxia. On the other hand, HIF-1 activation may contribute to the expression of DEC2 in osteosarcoma. This is the first demonstration of a novel DEC2-HIF-1 vicious cycle in osteosarcoma and a tumor-type specific role for DEC2.

**Electronic supplementary material:**

The online version of this article (doi:10.1186/s13046-015-0135-8) contains supplementary material, which is available to authorized users.

## Background

Osteosarcoma is the most common malignancy of bone, with a peak incidence in the second and third decades of life [1]. Osteosarcoma is very aggressive and has a high tendency to metastasis [2]. The 5-year event-free survival rate (EFS) for patients diagnosed with localized disease has improved since the invention of preoperative and postoperative chemotherapy. However, the prognosis for patients with metastatic osteosarcoma remains poor with a 5-year EFS of no more than 20% [3]. Currently, prognostic evaluation of osteosarcoma is mainly based on clinical parameters while useful molecular markers to predict tumor aggressiveness and to guide clinical treatment need to be explored [4].

The activation of hypoxia-inducible factor-1 (HIF-1) has been observed in osteosarcoma [5]; and clinically, osteosarcoma is commonly associated with abundant blood supply resulting from angiogenesis. Like other rapid growing solid tumors, osteosarcoma may develop a spatiotemporal hypoxic microenvironment during progression, hence activating HIF-1. HIF-1 is a heterodimeric transcription factor formed by HIF-1α and HIF-1β (also known as aryl hydrocarbon receptor nuclear translocator 1, ARNT1). HIF-1 is the main regulator of tumor angiogenesis, and regulates the expression of many genes relevant to oxygen transport, glucose metabolism, cell proliferation and apoptosis [6-8]. The protein level of HIF-1α is regulated by oxygen concentrations while HIF-1β is constitutively expressed. In addition to hypoxic activation of HIF-1α transactivation activity by inhibiting hydroxylation of the transactivation domain [9,10], hypoxic stabilization of HIF-1α is the other important mechanism for functional activation of HIF-1. Under normoxic conditions, oxygen-dependent hydroxylation of two prolyl residues of HIF-1α triggers a VHL (von Hippel Lindau)-mediated ubiquitination and proteasomal degradation of HIF-1α [6]. Hypoxia suppresses prolyl hydroxylase activity, preventing HIF-1α from hydroxylation and subsequent ubiquitination, thus stabilizing HIF-1α. Recent studies show that MDM2 might serve as an E3 ligase, facilitating VHL-independent proteasome-dependent degradation of HIF-1α [11,12]. Moreover, oncogenic signaling caused by either activation of oncogenes or loss of tumor suppressor functions may provide additional mechanisms to activate HIF-1 [6,13]. Increased HIF-1α levels have been found in many tumor types, accompanied by increased expression of HIF-1 target genes, including but not limited to, vascular endothelial growth factor A (VEGFA), phosphoglycerate kinase 1 (PGK1), angiopoietin-like 4 (ANGPTL4), carbonic anhydrase IX (CAIX) and hexose kinase 2 (HK2) [14]. HIF-1α overexpression has been correlated with high risk of metastasis and high mortality in many human cancers, including breast cancer and renal cell carcinoma [15-17], whereas the molecular mechanism underlying its overexpression in osteosarcoma and potential impact on osteosarcoma progression have not been fully understood.

Differentiated embryonic chondrocyte gene 2 (DEC2, also known as SHARP1 and bHLHE41) is a basic helix-loop-helix (bHLH) transcription regulator which has been implicated in regulating cell proliferation, apoptosis and circadian rhythms [18-22]. Recent studies have suggested that DEC2 may regulate HIF-1α expression in human breast and endometrial cancers [22-25]. In these reports, DEC2 was found to physically interact with and promote HIF-1α degradation, contributing to its suppressive effects on tumor progression and metastasis. However, it remains unknown whether, and how DEC2 affects HIF-1 activation in osteosarcomas.

In this study, we determined the expression levels of DEC2 and HIF-1α in osteosarcoma samples and analyzed the correlation between DEC2 expression and HIF-1α levels. We demonstrated that DEC2 knockdown in U2OS, MNNG and 143B cells led to down-regulation of HIF-1α protein levels and suppressed HIF-1-dependent activation of downstream target genes. By using an *in vitro* evolution model, we selected a highly invasive subpopulation (U2OS-M) from U2OS cells, and found that the highly invasive subpopulation had increased expression of DEC2 at both mRNA and protein levels, accompanied by accelerated HIF-1α accumulation upon hypoxia. Finally, we show that HIF-1 activation is sufficient to enhance DEC2 expression. Taken together, our data suggest that DEC2, which was shown to promote HIF-1α degradation in other tumors, may facilitate HIF-1 activation and metabolic reprograming in osteosarcomas, and that HIF-1 activation may, in turn, promote DEC2 expression, forming a vicious cycle.

## Materials and methods

### Human osteosarcoma samples

A total of 50 patients treated between 2006 and 2011 at the Department of Orthopedics, Shanghai Jiao Tong University Affiliated Sixth People’s Hospital (Shanghai, China) that were followed for 3 years were included in this study. All samples of human osteosarcoma were collected at the time of surgery. The study was approved by the Ethics Committee of Shanghai Jiao Tong University, and informed consent was obtained from all patients included in this study.

### Cell lines and cell culture

The MNNG and U2OS cell lines were purchased from the ATCC repository. 143B was a gift from Dr. M. King (Sydney Kimmel Cancer Center, Philadelphia) [26]. The cells were cultured in Dulbecco’s Modified Eagle Medium (DMEM) supplemented with 10% fetal bovine serum (FBS) (Biowest, South America Origin), 100 U/ml penicillin (Sigma–Aldrich) and 100 μg/ml streptomycin (Sigma–Aldrich) at 37°C in 5% CO_2_. The cells were regularly monitored to ensure that they were free of mycoplasma contamination. For hypoxic treatment, the cells were exposed to 1% O_2_ with 5% CO_2_ at 37°C for a duration indicated in each experiment with hypoxia chamber, or hypoxia Workstation (InVIVO2).

### Isolation of invasive subpopulation with trans-well chambers

The trans-well culture was performed as previously reported [27,28]. Briefly, 24-well plate inserts with 8-mm pore size chambers (Corning, USA) were used to isolate highly invasive subpopulation from the cultured U2OS parental cell line. First, cells were suspended in serum-free DMEM to a final cell density of 5 × 10^5^ cells/ml. 200 μl of cell suspension were seeded into the top chamber, which was coated with Matrigel. In the lower chamber, 800 μl of DMEM supplemented with 10% fetal bovine serum was added. Following incubation for 24 h at 37°C, the invasive cells on the underside of the membrane were expanded and used for subsequent rounds of selection. After six rounds of selection, the cell subpopulation able to migrate through the membranes was designated as U2OS-M.

### Synthetic small interfering RNAs (siRNAs) and RNA interference

The siRNA specifically targeting DEC2 (siDEC2) and negative control siRNA (siControl) were designed and synthesized by Biotend (Shanghai, China). The sequence used for siDEC2 was: 5′-ACGACACCAAGGAUACCUAdTdT/UAGGUAUCCUUGGUGUCGUdTdT-3′. Cells were transfected with siRNA using Lipofectamine 2000 (Invitrogen) by following the manufacturer’s protocol. For RNA extraction and western blotting assays, cells were used 48 h after transfection.

### RNA isolation and qRT–PCR assays

Total RNA was extracted from cultured cells using TRIzol reagent (Invitrogen, Carlsbad, CA, USA) according to the manufacturer’s protocol and quantified with Nanodrop 2000 (Thermo Fisher Scientific, Waltham, MA, USA). First-strand cDNA was synthesized with the PrimeScript RT Reagent Kit (TaKaRa, Shiga, Japan). Quantitative real-time polymerase chain reaction (qRT-PCR) was performed with SYBR Green premix Ex Taq (TaKaRa) with β-actin as an internal control. For all qRT-PCR analyses, the relative expression levels were compared to β-actin, and the unit was defined as 1/100,000 of β-actin level. The details of primers used for qRT-PCR are summarized in Additional file 1: Table S1.

### *In vitro* migration and invasion assays

Cell migration and invasion assays were performed in 24-well plates with 8-mm pore size chamber inserts (Corning, NY, USA), following established procedures. For migration assays, 5 × 10^4^ cells were placed into each well of the upper chamber with the non-coated membrane. For invasion assays, 1 × 10^5^ cells were placed into the upper chamber with the Matrigel-coated membrane, which was diluted with serum-free culture medium. In both assays, cells were suspended in 200 μl of DMEM without fetal bovine serum when they were seeded into the upper chamber. In the lower chamber, 800 μl of DMEM supplemented with 10% fetal bovine serum was added. After incubation for 16 h (migration assay) or 24 h (invasion assay) under normoxic or hypoxic conditions, the membrane inserts were removed from the plate, and non-invading cells were removed from the upper surface of the membrane. Cells that moved to the bottom surface of the chamber were fixed with 100% methanol for 20 min and stained with 0.1% crystal violet for 30 min. Then, at least 10 randomly selected fields were imaged and the cell numbers counted under a CKX41 inverted microscope (Olympus, Tokyo, Japan). The assays were conducted with at least three independent repeats.

### Western blotting analysis

Cell lysates were extracted from cultured cells with a mixture of T-PER Protein Extraction Reagent (Thermo Fisher Scientific), PhosSTOP (Roche, Basel, Switzerland) and Complete Mini (Roche). Protein samples were separated by 6% and 8% sodium dodecyl sulfate–polyacrylamide gel electrophoresis (SDS-PAGE) and transferred to nitrocellulose filter membranes (Millipore, Billerica, USA). After blocking in phosphate-buffered saline/Tween-20 containing 5% non-fat milk, the membranes were incubated with the following primary antibodies: DEC2 (Proteintech Group, Chicago, USA), HIF-1α (Proteintech Group, Chicago, USA), β-actin (Sigma-Aldrich). Horse radish peroxidase-conjugated anti-rabbit IgG (Sigma-Aldrich) was used as the secondary antibody. Subsequent visualization was detected with SuperSignal West Femto Maximum Sensitivity Substrate (Thermo Fisher Scientific).

### Immunohistochemistry (IHC)

Immunohistochemistry was performed with procedures similar to that described previously [29]. Briefly, formalin-fixed and paraffin-embedded sections were stained for rabbit anti-human HIF-1α polyclonal antibody (1:200; 20960-1-AP, Proteintech Group) and rabbit anti-human DEC2 polyclonal antibody (1:50; S8568, Sigma-Aldrich) using multi-use secondary antibody (1:1000; Dako, Ely, UK). Staining was visualized with the EnVision^TM^ HRP Rabbit/Mouse detection kit (Dako). All experimental procedures were performed according to the IHC staining protocol of corresponding manufacturers. Image acquisition was performed using an Olympus BX40 microscope with a 20× or 40× objective. Staining intensity was evaluated by systematically screening all slices and evaluating them according to an established 0–3 scale. The staining results for HIF-1α protein were classified as follows: 0, no staining; 1, nuclear staining in < 1% of cells; 2, nuclear staining in 1–10% of cells and/or weak cytoplasmic staining; 3, nuclear staining in > 10% of cells and/or distinct or strong cytoplasmic staining [30]. For DEC2 protein, the staining was evaluated with the same criteria. Samples graded as 0 and 1 were considered negative, and those graded as 2 and 3 were considered positive. Two triple-negative breast cancer (TNBC) lines known to have strong expression of DEC2 and HIF-1α [22] were used as positive controls. For negative controls, primary antibodies were substituted by PBS.

### Statistical analysis

All statistical analyses were performed using the Statistical Package for Social Sciences software (version 19.0) (SPSS, Inc., USA). Spearman’s rank correlation was used to determine the correlation between DEC2 and HIF-1α. Kaplan-Meier survival curve method was applied in the comparison of the Disease-free Survival between groups of HIF-1α positive and negative expressions. Data were plotted with GraphPad Prism 5 software (GraphPad Software, Inc, La Jolla, CA, USA). Quantitative variables were presented as means with standard error of mean (s.e.m.), unless otherwise noted, and analyzed by Student’s *t*-test between two groups (two-tailed; P < 0.05 was considered statistically significant).

## Results

### Expression of DEC2 and HIF-1α in human osteosarcoma specimens correlates with poor prognosis

**Figure 1:**
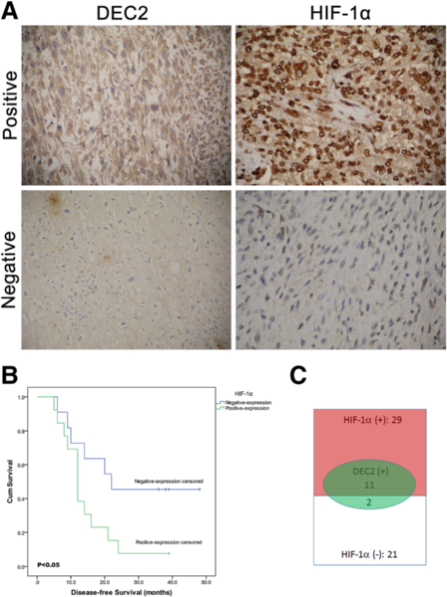
**HIF-1α levels in human osteosarcoma are correlated to disease-free survival (DFS).** **A**. Paraffin-embedded osteosarcoma tissues were retrieved and deparaffinized and used in immunohistochemical studies. The pathological diagnosis was re-confirmed. Representative micrographs for immunohistochemical staining of HIF-1α (right) and DEC2 (left) were shown. Magnification: 40 ×. **B**. Kaplan-Meier plot showing DFS in osteosarcoma patients, stratified according to positive or negative expression levels of HIF-1α. Primary osteosarcoma tissues with scale 2 and 3 of HIF-1α expression were classified as HIF-1α positive, scale 0 and 1, negative. **C**. A diagram showing the distribution of DEC2 positive and HIF-1α positive cases in osteosarcoma samples, showing a striking positive correlation.

To investigate the involvement of HIF-1α and DEC2 in the progression and metastasis of osteosarcoma, we used a cohort of 50 primary osteosarcoma samples obtained from clinical patients. Immunohistochemistry results showed that 29 cases were positive for HIF-1α (58%) and 13 specimens were positive for DEC2 (26%) (Figure 1A). Among these 50 cases, 24 cases of osteosarcoma were followed up successfully. Using Kaplan-Meier survival analysis (Figure 1B), we found that high expression of HIF-1α was correlated with higher probability of metastasis and significantly reduced disease-free survival (DFS), suggesting that HIF-1 activation may be a determining factor for metastasis and EFS in osteosarcoma.

Since DEC2 has been reported as a regulator of HIF-1α stability in breast and endometrial cancers [22,23], we next asked if DEC2 negatively correlates with HIF-1 activation in osteosarcoma. We surprisingly found that among the 13 DEC2 positive cases, 11 were also HIF-1α positive. Statistical analysis revealed a strong positive correlation between DEC2 and HIF-1α expression in osteosarcomas (Table 1, Spearman’s rank correlation, ** P < 0.01). These findings suggest a positive role of DEC2 in HIF-1α stability in osteosarcoma, which may be different from that reported in other types of tumors.

**Table 1:** **Correlation between the expression of DEC2 and HIF-1α in human osteosarcomas**

**DEC2**	HIF-1α expression scale*				**Total**
	**0**	**1**	**2**	**3**	
0	0 (0%)	4 (8%)	2 (4%)	1 (2%)	7 (14%)
1	2 (4%)	13 (26%)	11 (22%)	4 (8%)	30 (60%)
2	0 (0%)	2 (4%)	2 (4%)	7 (14%)	11 (22%)
3	0 (0%)	0 (0%)	0 (0%)	2 (4%)	2 (4%)
Total	2 (4%)	19 (38%)	15 (30%)	14 (28%)	50 (100%)

Spearman’s rank correlation, P < 0.01, r_s_ = 0.425.

*0-3 scale represents the staining intensity as classified in the Materials and Methods.

### DEC2 facilitates HIF-1α expression in osteosarcoma cell lines

**Figure 2:**
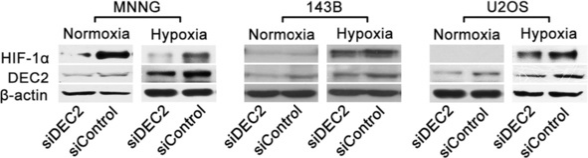
**The effects of DEC2 knockdown on HIF-1α protein levels in osteosarcoma cell lines.** DEC2 was knocked down in U2OS, MNNG and 143B cell lines: Cells were transfected with indicated siRNAs and cultured for 48 h under either normoxia or hypoxia prior to cell harvest and protein sample collection. HIF-1α and DEC2 levels were determined by Western blotting, and β-actin was determined for normalization of sample loading.

To further investigate the functional interaction between DEC2 and HIF-1α, we synthesized siDEC2 and negative control siRNA (siControl). Using these siRNA, we knocked down the expression of DEC2 in osteosarcoma cell lines U2OS, MNNG and 143B, followed by exposing the cells to either normoxia or hypoxia. Western blotting analysis revealed that under normoxic conditions HIF-1α was expressed in MNNG and 143B cells, but was not detectable in U2OS cells (Figure 2). DEC2 knockdown did not affect HIF-1α mRNA levels (Additional file 2: Figure S1 and data not shown). However, DEC2 knockdown effectively down-regulated HIF-1α protein levels in MNNG and 143B cell lines under normoxic conditions (Figure 2), and in all three cell lines under hypoxic conditions, indicating that in osteosarcoma cells DEC2 positively regulates HIF-1α at the post-transcriptional levels.

### DEC2 is required for optimal HIF-1α stabilization and HIF-1 activation upon hypoxia

**Figure 3:**
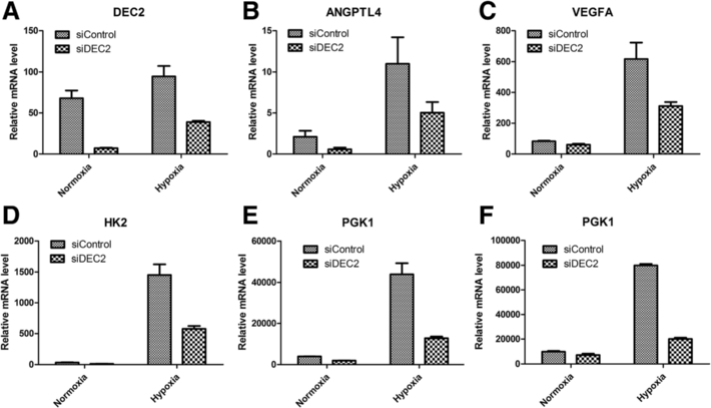
**DEC2 is required for HIF-1α transactivation activity in U2OS.** Cells were transfected with either control (NC) or DEC2 siRNAs and exposed to either 21% or 1% oxygen for 8 h.** A**. Confirmation of DEC2 knockdown by qRT-PCR. **B-F**. Analyses of the expression levels of HIF-1α downstream target genes ANGPTL4 **(B)**, VEGFA **(C)**, HK2 **(D)**, PGK1 **(E)** in U2OS cells and PGK1 **(F)** in MNNG cells by qRT-PCR.

Under hypoxia or when driven by oncogenic signaling, HIF-1α levels are stabilized to activate a set of target genes, such as angiopoietin-like 4 (ANGPTL4), vascular endothelial growth factor A (VEGFA) and hexokinase 2 (HK2), which promote adaptations at cellular, tissue and organismal levels [6,31,32]. In this study, we also found that hypoxia promoted U2OS cell invasiveness and migration (Additional file 3: Figure S2). We asked if DEC2 knockdown impairs hypoxia-stimulated HIF-1α accumulation and HIF-1-dependent gene expression. We knocked down DEC2 expression in U2OS cells which have undetectable basal levels of HIF-1α under normoxic condition, and cultured them under either normoxic or hypoxic condition for 48 h. Using qRT-PCR, we confirmed that the siRNA efficiently knocked down DEC2 (Figure 3A, P > 0.05), and that DEC2 knockdown did not affect HIF-1α mRNA levels (Figure 1). The mRNA levels of ANGPTL4, VEGFA, HK2 and PGK1, four classical HIF-1 downstream target genes, were stimulated by hypoxia in U2OS cells; however, DEC2 knockdown partially suppressed the hypoxia-stimulated expression of these HIF-1 target genes (Figures 3B-E). Similar results were obtained in MNNG and 143B cells (Figure 3F and Additional file 4: Figure S3). These results indicate a distinctive role of DEC2 in osteosarcoma, which facilitates HIF-1 activation and is apparently different from those observed in other types of tumors [22,23].

### Increased expression of DEC2 and HIF-1α is associated with increased invasiveness and metastatic potential of osteosarcomas

**Figure 4:**
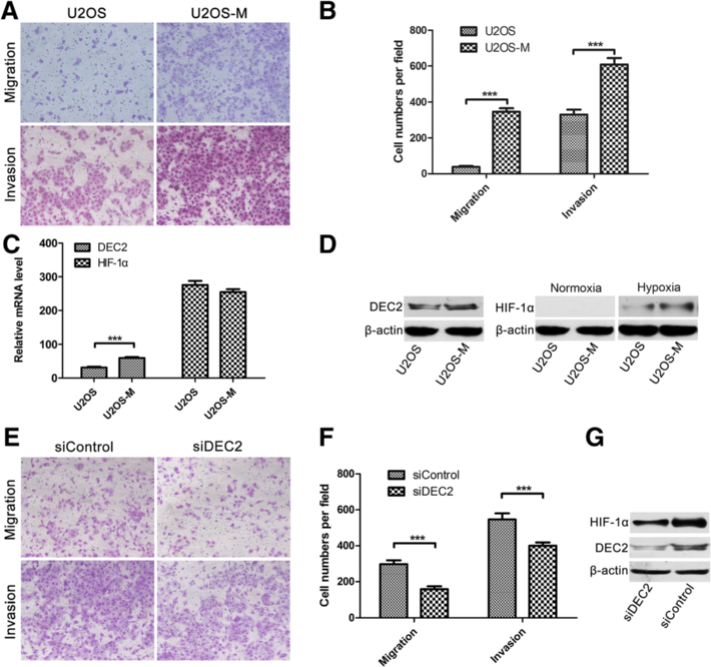
**Invasive U2OS-M cells have higher levels of DEC2 and accumulate HIF-1α more rapidly in response to hypoxia. A**. *In vitro* migration and invasion activity of U2OS and U2OS-M. Migration and invasion activities were measured *in vitro* with trans-well chambers, as described in Materials and Methods. Photos are representative fields of invasive cells on the membrane. Magnification: 100 ×. **B**. Quantification of data shown in A. Bar graphs represent the average number of cells on the underside of the membrane (means ± s.e.m.) **C**. U2OS-M cells express higher level of DEC2 mRNA than the parental U2OS, while the HIF-1α mRNA level was not significantly affected as determined by qRT-PCR (P > 0.05). **D**. U2OS-M cells express higher level of DEC2 protein than U2OS cells. Cells were cultured under regular culture conditions or hypoxic condition (1 h) prior to harvest. Protein levels were determined by Western blotting. Under regular culture conditions, neither U2OS nor U2OS-M showed detectable HIF-1α. After 1 h hypoxic exposure, U2OS-M cells, which express higher level of DEC2, accumulated more HIF-1α than U2OS. **E**. U2OS-M Cells were transfected with either control or DEC2 siRNAs for 48 h**.** Migration and invasion activities were measured *in vitro* with trans-well chambers, as described in Materials and Methods. Photos are representative fields of invasive cells on the membrane. Magnification: 100 ×. **F**. Quantification of data shown in **E**. Bar graph presents the average numbers of cells on the underside of the membrane (means ± s.e.m.). **G**. DEC2 knockdown in U2OS-M slowed down HIF-1α accumulation. Cells were transfected with indicated siRNAs and cultured for 48 h. Cells were exposed to hypoxia for 1 h prior to protein sample collection. HIF-1α and DEC2 levels were determined by Western blotting, and β-actin was determined for normalization of loading. ***: P < 0.001.

To further explore the role of DEC2 and HIF-1α in osteosarcoma progression, we used the repetitive trans-well approach [27,33] to select a more invasive subpopulation from the parental U2OS cells. After a total of six rounds of selection, we obtained an invasive subpopulation named U2OS-M. As shown in Figure 4A and B, the migration and invasion ability of U2OS-M cells were significantly higher than that of the parental U2OS cells. We next asked if the increased invasiveness and migration potential are associated with HIF-1 activation and DEC2 expression. We found that the mRNA levels of HIF-1α were not changed, but that the mRNA levels of DEC2 were significantly higher in U2OS-M than U2OS (Figure 4C). Consistent with the mRNA levels, the protein levels of DEC2 in U2OS-M cells were also remarkably higher than that in U2OS (Figure 4D, left part *** P < 0.001). To investigate if increased expression of DEC2 affects the hypoxic stabilization of HIF-1α, we treated both U2OS and U2OS-M with hypoxia for 1 h. Western blotting indicated that under normoxic conditions, neither U2OS nor U2OS-M had detectable HIF-1α protein. However, after hypoxic treatment, U2OS-M cells accumulated HIF-1α more rapidly than the parental U2OS cells (Figure 4D, right part). The role of DEC2 was further confirmed when DEC2 knockdown in U2OS-M cells impeded cell invasiveness and migration (Figure 4E and F, *** P < 0.001) and slowed down HIF-1α accumulation (Figure 4G). These results indicate that DEC2 may contribute to the invasiveness of osteosarcoma by facilitating hypoxic HIF-1α accumulation.

### HIF-1 activation is sufficient to upregulate DEC2 in osteosarcomas

**Figure 5:**
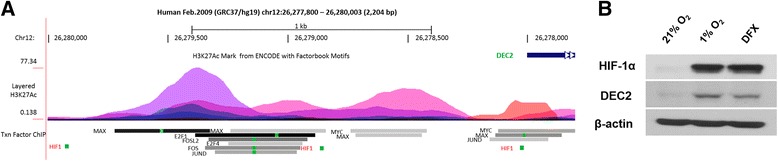
**HIF-1 activation is sufficient to upregulate DEC2 in osteosarcomas under normoxic condition. A**. Bioinformatics analysis of DEC2 promoter reveals that a region with highly acetylated histone H3 at K27 contains potential binding sites for multiple transcription regulators including HIF-1, c-Fos/Jun, c-Myc/Max and E2F. **B**. HIF-1 activation is sufficient to upregulate DEC2 levels under normoxic condition. 143B cells were cultured in hypoxia workstation or treated with 100 μM of DFX for 6 h prior to harvest to stabilize HIF-1α. Western blots show HIF-1α stabilization up-regulates DEC2.

Finally, we asked how DEC2 expression is regulated in osteosarcomas. We performed a bioinformatics analysis of the DEC2 promoter, seeking transcription factors that could potentially interact with the DEC2 promoter. The 5,045 bp region encoding the DEC2 gene is located on the reverse strand Chromosome 12: 26,272,959-26,278,003 in GRCh37 coordinates. With the UCSC Genome Browser (http://genome.ucsc.edu/), the 2,000 bp upstream of the DEC2 transcription initiation site (chr12:26,278,804-26,280,003) was found to contain regions associated with highly acetylated histone H3 at lysine 27 (H3K27Ac) which indicates transcription regulator binding sites. Layered H3K27Ac is shown in Figure 5A, and Transcription Factor ChIP (Txn Factor ChIP) was chosen to evaluate the regulatory elements based on ENCODE Project (https://www.encodeproject.org/). Due to the limited number of transcription factors included in ENCODE Project, PROMO (http://alggen.lsi.upc.es/) was also utilized to predict transcription regulators of DEC2. From these analyses, binding sites for ninety seven transcription factors were predicted within a dissimilarity lesser than 5%. Particularly, the analyses revealed that one H3K27Ac region contains putative sites for c-Fos/Jun and c-Myc/Max (Figure 5A), two transcription factors encoded by oncogenes that are frequently activated in osteosarcomas [34]. This region also contains putative binding sites for E2F, a family of cell cycle regulatory transcription factors controlled by the retinoblastoma family of tumor suppressors (Rb) [35]. Interestingly, the analyses revealed three potential HIF-1 binding sites located at chr12:26,278,014-26,278,022 (TCCGCACGT), chr12:26,278,851-26,278,859 (AAAGCACGT) and chr12:26,279,908-26,279,916 (ACGTGCCCT) (Figure 5A). A review of our qRT-PCR data revealed that hypoxia increased DEC2 mRNA levels (Figure 3A). To confirm whether HIF-1 activation is sufficient to upregulate DEC2, we treated 143B cells with desferrioxamine (DFX), a prolyl-hydroxylase inhibitor which stabilizes HIF-1α under normoxic conditions [13]. As shown in Figure 5B, either hypoxia or DFX treatment was able to stabilize HIF-1α; the normoxic stabilization of HIF-1α by DFX exposure effectively upregulated DEC2 expression, indicating HIF-1 activation is sufficient to enhance DEC2 expression in osteosarcomas.

## Discussion

As the master regulator of glucose utilization and tissue angiogenesis, HIF-1 is activated in many solid tumors during tumor progression to sustain energy metabolism and biosynthesis [36], and to promote tumor cell migration [37]. In addition to hypoxia, oncogene activation and loss of tumor suppressor genes’ function may contribute to normoxic activation of HIF-1 [13,34,35,38]. Glucose transporter 1 (GLUT1), HK2, ANGPTL4 and VEGF are well known downstream target genes of HIF-1 and are associated with tumor specific metabolic reprogramming, angiogenesis and metastasis. In this study, we demonstrated that HIF-1α positivity predicts an unfavorable prognosis of osteosarcomas. Considering the previous reports that DEC2 promoted HIF-1α degradation in breast and endometrial cancers, one would expect a negative correlation between DEC2 and HIF-1α. Surprisingly, our clinical data based on studies on 50 osteosarcoma samples revealed that DEC2 expression levels have a positive relationship with HIF-1α levels, raising the possibility that DEC2 overexpression predicts a poor prognosis of osteosarcoma, and may contribute to the development and progression of osteosarcomas. Indeed, we observed that HIF-1 target genes ANGPTL4, VEGFA, GLUT1, PGK1 and HK2 were down-regulated at mRNA level when DEC2 is knocked down in several osteosarcoma cell lines, while the mRNA level of HIF-1α showed no obvious change by the DEC2 knockdown. Since HIF-1α levels are mainly regulated at the level of protein stability [39], and HIF-1α is remarkably down-regulated with the depletion of DEC2, our data suggest that DEC2 may be a facilitator for HIF-1α stabilization in osteosarcomas under the influence of oncogenic signaling or in a hypoxic tumor microenvironment.

DEC2 is expressed in various embryonic and adult tissues, functioning as a transcription repressor of E-box activity [18,19]. Emerging evidence suggests that it suppresses tumor progression in oral, endometrial and breast cancers, and mechanistically, a role in apoptosis, proliferation and metastasis has been proposed [22,23,25]. Generally, these previous studies suggested that DEC2 functions as a tumor suppressor. In this study, in 24 cases that were followed up to 3 years, high expression of HIF-1α was shown to indicate a significantly higher probability of developing metastasis and of reduced DFS. Intriguingly, the expression level of DEC2 is positively correlated with HIF-1α. In addition, the expression of DEC2 indicates a tendency for poor prognosis. However, based on the current sample size, our available data failed to meet the criteria of statistics significance. A larger sample size is required to further examine the relationship between DEC2 expression and the prognosis of osteosarcomas.

Using an *in vitro* evolution model, we isolated a more invasive subpopulation (U2OS-M) from the human osteosarcoma U2OS cell line, which has been extensively used to investigate osteosarcoma metastasis [27,28,40]. Our data showed that the subpopulation selected from repetitive trans-well culture procedures has enhanced invading and metastasizing capabilities. Interestingly, compared with the parental U2OS cell line, the more invasive U2OS-M population has higher levels of DEC2. Whereas no HIF-1α protein in either U2OS or U2OS-M cells under normal culture conditions was observed, U2OS-M cells accumulated HIF-1α more rapidly than the parental U2OS cells after exposure to hypoxia, indicating the enhanced expression of DEC2 sensitizes osteosarcoma cells to hypoxia. The role of DEC2 in HIF-1 activation and invasiveness of osteosarcomas was further confirmed by DEC2 knockdown in U2OS-M lowered the fast hypoxic accumulation of HIF-1α and the invasiveness.

Mechanistically, the reduction of HIF-1α levels mediated by DEC2 knockdown was blocked in the presence of a proteasome inhibitor (data not shown), suggesting that lack of DEC2 function promotes the proteasome-dependent degradation of HIF-1α. On the other hand, enhanced DEC2 may partially suppress the VHL-independent, ubiquitination-independent degradation pathway [41,42]. This is apparently the opposite of the observation from breast cancers, where DEC2 promoted VHL-independent degradation of HIF-1α. While the detailed mechanism underlying the DEC2-facilitated stabilization of HIF-1α in osteosarcomas remains to be further explored, our results indicate that DEC2 could promote osteosarcoma invasiveness and metastasis by enhancing HIF-1 function.

While the transcriptional regulation of DEC2 expression may be complicated and affected by oncogenic signaling pathways (Figure 5A), we observed that DEC2 levels were enhanced by hypoxia in the osteosarcoma cell lines we studied. Importantly, stabilization of HIF-1α is sufficient to enhance DEC2 protein levels, and inhibiting prolyl hydroxylase activity by DFX under normoxic condition also enhanced DEC2 levels (Figure 5B). Consistent with our observation, an early study reported that DEC2 promoter possesses a hypoxia responsive element between −863 and -258 bp (covering the predicted HIF-1 binding site at chr12:26,278,851-26,278,859), which physically bound HIF-1 in electrophoretic mobility shift assays [43]. Since multiple oncogenic signaling pathways may be activated thus triggering normoxic HIF-1 activation in osteosarcomas, it is very possible that the oncogenic activation of HIF-1 plays an important role in hypoxia-independent up-regulation of DEC2. It is also possible that the activation of c-Fos/Jun, c-Myc/Max or loss of Rb family tumor suppressors is able to trigger the up-regulation of DEC2, which facilitates HIF-1 activation upon hypoxia. As such, either case may provide an explanation for normoxic HIF-1 activation and DEC2 up-regulation in osteosarcoma. Further studies are needed to investigate these possibilities.

**Figure 6:**
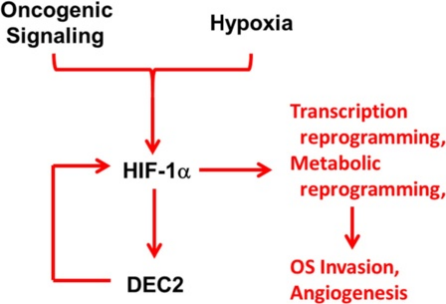
**Proposed model showing the intriguing functional DEC2-HIF-1 association in the progression of osteosarcoma.** In osteosarcomas, a combination of hypoxia and oncogenic signaling may increase the levels of HIF-1α, which upregulates DEC2 expression. DEC2 in turn facilitates the stabilization of HIF-1α, sensitizing osteosarcoma cells to hypoxic or oncogenic signaling and rapidly activating HIF-1 function.

Taking together, we propose a HIF-1-DEC2 vicious cycle model in osteosarcoma (Figure 6). In this model, oncogenic signaling or spatiotemporal hypoxia in the osteosarcoma microenvironment stabilizes HIF-1α and activates HIF-1. HIF-1 transcriptionally up-regulates DEC2, which in turn facilitates HIF-1 activation. This deregulated HIF-1 activation eventually contributes to the transcriptional reprograming, metabolic reprograming, angiogenesis and invasive nature of osteosarcomas.

As discussed above, DEC2 overexpression promotes HIF-1α degradation in breast and endometrial cancers. It is particularly interesting, but remains unclear, why DEC2 functions differently in osteosarcomas. One possibility is that osteosarcomas have a dominant negative form of DEC2, which may have arosen by mutation, differential splicing or other mechanism. Since the molecular weight of DEC2 detected in osteosarcomas by Western blotting is very similar to that seen in other types of tumors, large deletion or truncation of DEC2 in osteosarcomas is unlikely. The mutation, if any, would be more likely to be in the form of a point mutation leading to an amino acid substitution. Whether osteosarcomas have a mutated form of DEC2 remains to be investigated by cloning and sequencing of the DEC2 gene from osteosarcomas, which is currently under way. It is also possible that DEC2 forms a protein complex that regulates HIF-1α stability; stabilizing or destabilizing of HIF-1α is determined by the association or dissociation with other components of the complex, or a change of subcellular localization regulated by the complex dynamics. Further studies are needed to decipher the molecular mechanisms underlying this intriguing mystery.

## Conclusion

In contrast to other tumors, osteosarcoma shows that DEC2 expression positively correlates with HIF-1α levels, and that high HIF-1α expression levels predict poor prognosis. While DEC2 facilitates HIF-1α stabilization, HIF-1 activation also upregulates DEC2 expression, forming a vicious cycle in osteosarcoma. The DEC2-HIF-1 vicious cycle may be triggered by either oncogenic signaling or the hypoxic microenvironment in osteosarcomas and so contribute to metabolic reprogramming and invasiveness. This provides a new prognostic biomarker and therapeutic target for highly invasive and metastatic osteosarcomas.

## Additional files

Additional file 1: Table S1. Primers Used in qRT-PCR. Additional file 2: Figure S1. Hypoxia enhances U2OS cell migration *in vitro*. Human U2OS osteosarcoma cells were exposed to either 21% or 1% oxygen. Cell migration and invasion were assessed as described in Materials and Methods. A. Representative photographs of migrated cells on the membrane at a magnification of 100 ×. B. Data are presented as the means ± s.e.m. of triplicate samples, and are representative of three independent experiments. * P < 0.05, ** P < 0.01. Additional file 3: Figure S2. DEC2 knockdown does not affect the mRNA levels of HIF-1α in U2OS under either normoxic or hypoxic conditions. Cells were transfected with either control (siControl) or DEC2 (siDEC2) siRNAs and exposed to either 21% or 1% oxygen levels for 8 h. Total RNA were extracted, and the mRNA levels of HIF-1α were quantitated by qRT-PCR. Additional file 4: Figure S3. DEC2 knockdown blunts the hypoxic stimulation of HIF-1 target genes GLUT1 and CAIX. Osteosarcoma cell line 143B was transfected with control or DEC2 siRNAs and cultured for 48 h. Then the cells were exposed to either 21% or 1% oxygen (In VIVO2, Ruskinn Technology Limited, UK) for 8 h and cells were harvested and total RNA extracted. The expression levels of GLUT1 and CAIX (CA9) were determined by quantitative RT-PCR with Taqman gene expression primers (Invitrogen) and StepOnePlus instrument (Applied Biosystems), and the mRNA levels were presented in relative quantity (RQ).

## Abbreviations

HIF-1: Hypoxia-inducible factor 1
DEC2: Differentiated embryonic chondrocyte gene 2
EFS: Event-free survival rate
ARNT1: Aryl hydrocarbon receptor nuclear translocator 1
MDM2: MDM2 proto-oncogene, E3 ubiquitin protein ligase
VEGFA: Vascular endothelial growth factor A
PGK1: Phosphoglycerate kinase 1
ANGPTL4: Angiopoietin-like 4
CAIX (CA9): Carbonic anhydrase IX
HK2: Hexose kinase 2
SHARP1: Enhancer-of-split and hairy-related protein 1
bHLHE41: basic helix-loop-helix family, member e41
IHC: Immunohistochemistry
TNBC: Triple-negative breast cancer
DFS: Disease-free survival
H3K27Ac: Acetylated histone H3 at lysine 27
Txn Factor ChIP: Transcription Factor ChIP
DFX: Desferrioxamine
GLUT1: Glucose transporter 1
