# Supplementary material for: DEC2 expression is positively correlated with HIF-1 activation and the invasiveness of human osteosarcomas
Source: J Exp Clin Cancer Res. 2015 Feb 28;34(1):22. doi: 10.1186/s13046-015-0135-8 (PMC4379712; doi:10.1186/s13046-015-0135-8)
Supplement: Additional file 1: Table S1. — Primers Used in qRT-PCR. [file 13046_2015_135_MOESM1_ESM.pdf]

Supplementary Table: Primers Used in qRT-PCR

Name	Sequence
DEC2	5’-CGAGACGACACCAAGGATACC-3’
	5’-TTCTGATGCTGTTGCTCGGT-3’
HIF-1α	5’-GCCAGACGATCATGCAGCTA-3’
	5’-ATCCATTGATTGCCCCAGCA-3’
ANGPTL4	5’-ACCTCAGATGGAGGCTGGAC-3’
	5’-CCCGTGATGCTATGCACCTT-3’
VEGFA	5’-CTCCACCATGCCAAGTGGTC-3’
	5’-GCAGTAGCTGCGCTGATAGA-3’
HK2	5’-GCTGCAAGAGGAATGAGGCT-3’
	5’-TGGCTACTTACAACCGAAGCA-3’
PGK1	5’-TTGACCGAATCACCGACCTC-3’
	5’-AGCAGCCTTAATCCTCTGGT-3’
β-Actin	5’-GTCATTCCAAATATGAGATGCGT-3’
	5’-GCATTACATAATTTACACGAAAGCA-3’
