# Supplementary figures and images for: DEC2 expression is positively correlated with HIF-1 activation and the invasiveness of human osteosarcomas
Source: J Exp Clin Cancer Res. 2015 Feb 28;34(1):22. doi: 10.1186/s13046-015-0135-8 (PMC4379712; doi:10.1186/s13046-015-0135-8)

# HIF-1 $\alpha$

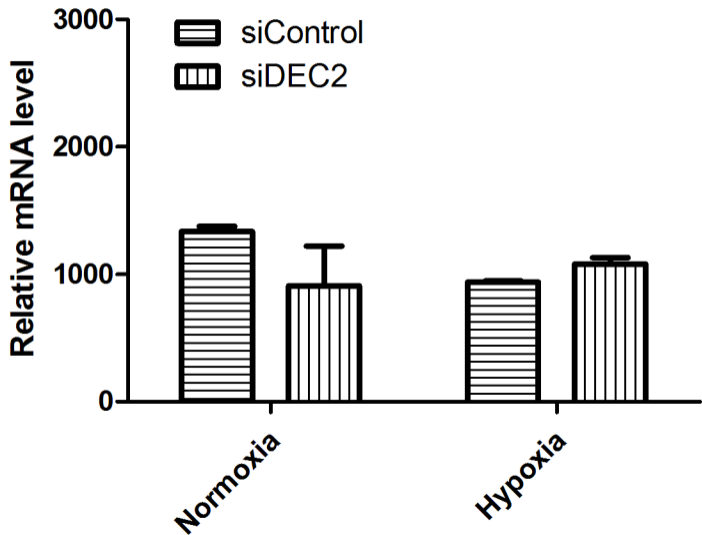

Supplement: Additional file 2: Figure S1. — Hypoxia enhances U2OS cell migration in vitro. Human U2OS osteosarcoma cells were exposed to either 21% or 1% oxygen. Cell migration and invasion were assessed as described in Materials and Methods. A. Representative photographs of migrated cells on the membrane at a magnification of 100 ×. B. Data are presented as the means ± s.e.m. of triplicate samples, and are representative of three independent experiments. * P < 0.05, ** P < 0.01. [file 13046_2015_135_MOESM2_ESM.pdf]

**A**

Normoxia

Hypoxia

Migration

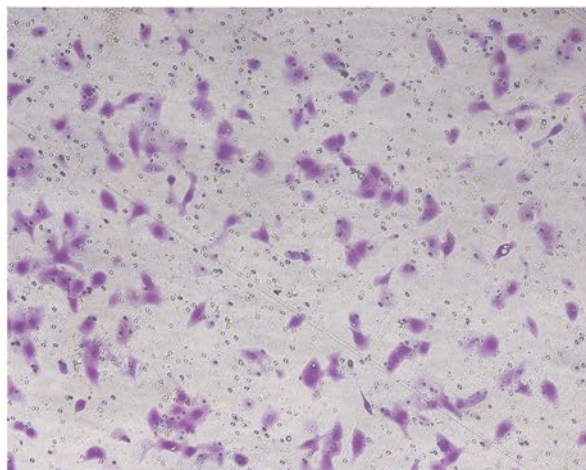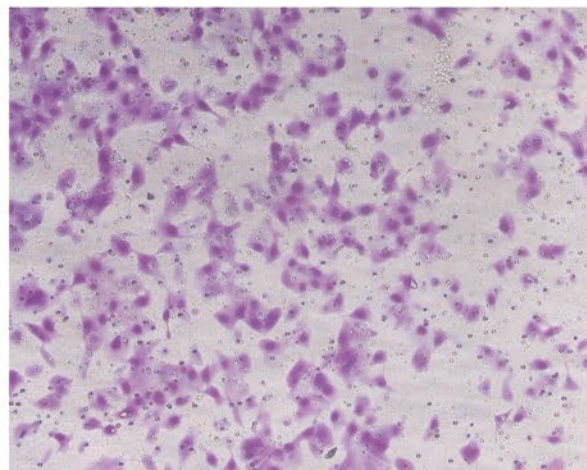

Invasion

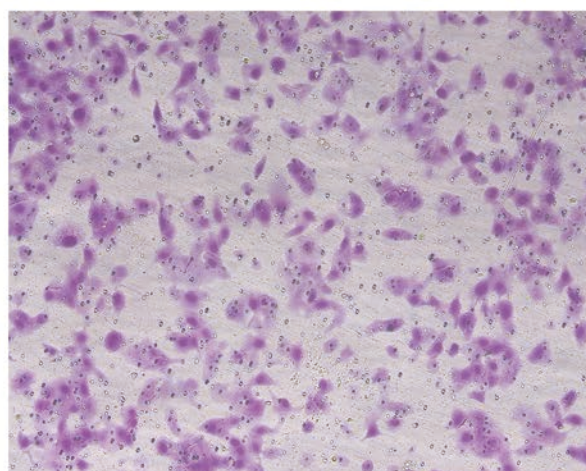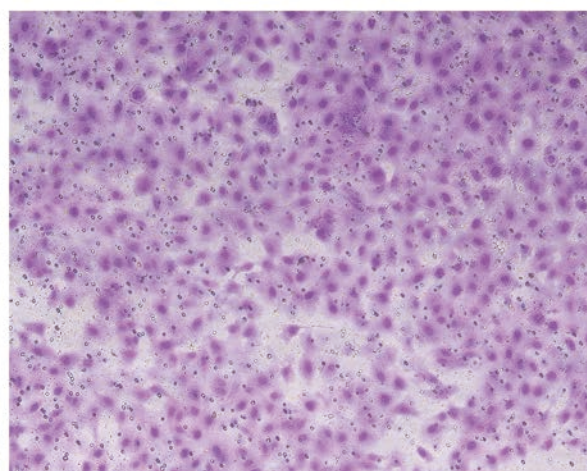**B**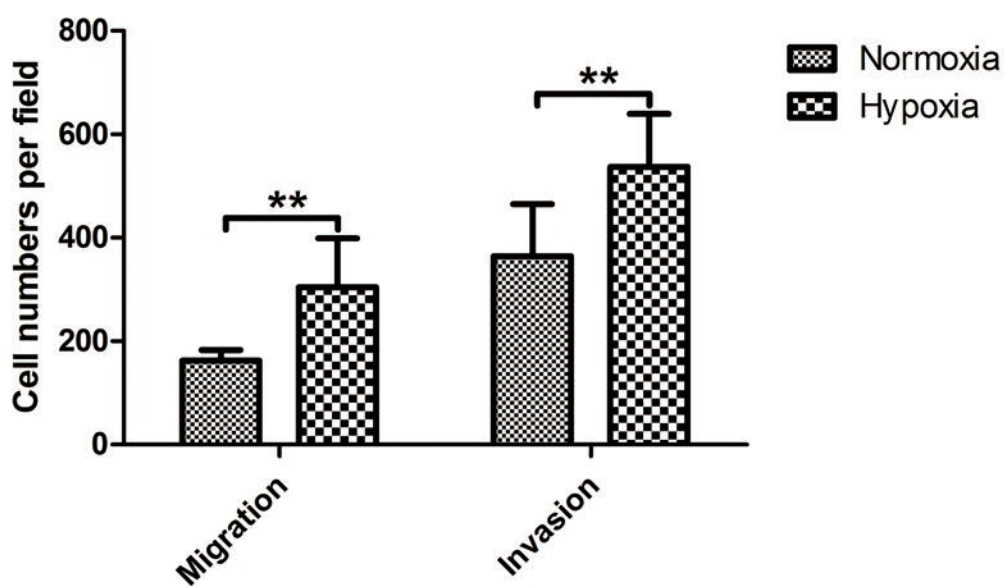

Supplement: Additional file 3: Figure S2. — DEC2 knockdown does not affect the mRNA levels of HIF-1α in U2OS under either normoxic or hypoxic conditions. Cells were transfected with either control (siControl) or DEC2 (siDEC2) siRNAs and exposed to either 21% or 1% oxygen levels for 8 h. Total RNA were extracted, and the mRNA levels of HIF-1α were quantitated by qRT-PCR. [file 13046_2015_135_MOESM3_ESM.pdf]
