# Supplementary material for: DEC2 expression is positively correlated with HIF-1 activation and the invasiveness of human osteosarcomas
Source: J Exp Clin Cancer Res. 2015 Feb 28;34(1):22. doi: 10.1186/s13046-015-0135-8 (PMC4379712; doi:10.1186/s13046-015-0135-8)
Supplement: Additional file 4: Figure S3. — DEC2 knockdown blunts the hypoxic stimulation of HIF-1 target genes GLUT1 and CAIX. Osteosarcoma cell line 143B was transfected with control or DEC2 siRNAs and cultured for 48 h. Then the cells were exposed to either 21% or 1% oxygen (In VIVO2, Ruskinn Technology Limited, UK) for 8 h and cells were harvested and total RNA extracted. The expression levels of GLUT1 and CAIX (CA9) were determined by quantitative RT-PCR with Taqman gene expression primers (Invitrogen) and StepOnePlus instrument (Applied Biosystems), and the mRNA levels were presented in relative quantity (RQ). [file 13046_2015_135_MOESM4_ESM.pdf]

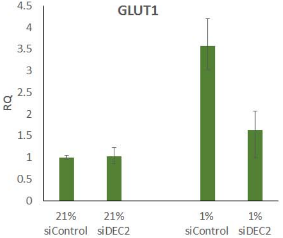

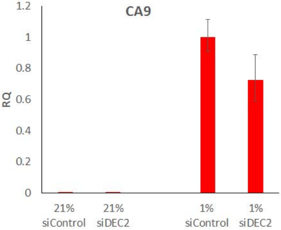

GLUT1	RQ	RQ min	RQ max
21% siControl	1	0.952	1.051
21% siDEC2	1.019	0.847	1.225
1% siControl	3.566	3.029	4.197
1% siDEC2	1.626	0.993	2.061

CA9	RQ	RQ min	RQ max
21% siControl	NA	NA	NA
21% siDEC2	NA	NA	NA
1% siControl	1	0.908	1.113
1% siDEC2	0.725	0.591	0.889
